# Insecticide susceptibility of natural populations of *Anopheles coluzzii and Anopheles gambiae* (*sensu stricto*) from Okyereko irrigation site, Ghana, West Africa

**DOI:** 10.1186/s13071-016-1462-0

**Published:** 2016-03-31

**Authors:** Joseph Chabi, Philip K. Baidoo, Alex K. Datsomor, Dora Okyere, Aikins Ablorde, Alidu Iddrisu, Michael D. Wilson, Samuel K. Dadzie, Helen P. Jamet, Joseph W. Diclaro

**Affiliations:** Vestergaard-NMIMR Vector Labs (VNVL), Noguchi Memorial Institute for Medical Research, University of Ghana, Legon, Accra, Ghana; Parasitology Department, Noguchi Memorial Institute for Medical Research, University of Ghana, Legon, Accra, Ghana; Department of Theoretical and Applied Biology, Kwame Nkrumah University of Science and Technology, Kumasi, Ghana; Vestergaard, Washington, DC USA; Vector Biology Research Program, U.S. Naval Medical Research Unit No 3, Cairo, Egypt

**Keywords:** Insecticide resistance, Malaria, *An. gambiae* s.s, *An. coluzzii*, Irrigation

## Abstract

**Background:**

The increasing spread of insecticide resistance in malaria vectors has been well documented across sub-Saharan Africa countries. The influence of irrigation on increasing vector resistance is poorly understood, and is critical to successful and ethical implementation of food security policies. This study investigated the insecticide resistance status of *An. gambiae* (*s.l.*) mosquitoes collected from the irrigated rice area of Okyereko, a village containing about 42 hectares of irrigated field within an irrigation project plan in the Central Region of Ghana. Large amounts of insecticides, herbicides and fertilizers are commonly used in the area to boost the annual production of the rice.

**Methods:**

Mosquito larvae were collected and adults were assayed from the F_1_ progeny. The resistance status, allele and genotype were characterized using WHO susceptibility testing and PCR methods respectively.

**Results:**

The *An. gambiae* (*s.l.*) populations from Okyereko are highly resistant to DDT and pyrethroid insecticides, with possible involvement of metabolic mechanisms including the elevation of P450 and GST enzyme as well as P-gp activity. The population was mostly composed of *An. coluzzii* specimens (more than 96 %) with *kdr* and *ace-1* frequencies of 0.9 and 0.2 %, respectively.

**Conclusion:**

This study brings additional information on insecticide resistance and the characterization of *An. gambiae* (*s.l.*) mosquitoes from Okyereko, which can be helpful in decision making for vector control programmes in the region.

## Background

The spread of insecticide resistance is threatening the continued efficacy of current malaria control tools and is exacerbated by the use of insecticides in controlling both agricultural pests and vectors that affect public health [1–5]. The Republic of Ghana is not exempt from this trend [6–8]. Pyrethroid resistance in malaria vectors is increasingly reported from different parts of Africa, and has been associated with selection pressure resulting from the scaling up of insecticide treated materials such as Long Lasting Insecticidal Nets (LLINs) and indoor residual house spraying (IRS) [9, 10], as well as the application of agricultural pesticides [1, 3, 11].

The dominant mosquito species responsible for the transmission of malaria parasites in Africa includes *An. gambiae* Giles (*s.s.*) and *An. coluzzii* Coetzee & Wilkerson (formerly *An. gambiae* S and M molecular forms, respectively), *An. arabiensis* Patton and *An. funestus * Giles (*s.s.*), all of which are widespread throughout tropical and subtropical Africa. *Anopheles arabiensis* prefers drier habitats and *An. coluzzii* is restricted to West-Central Africa [12, 13]. The adult behaviors and larval biology of these species are different, which impact the efficacy and/or suitability of control measures. Ecological differentiation indicates that *An. coluzzii* preferentially exploits immature sites that exist across seasons and are more associated with human activities, such as those created by irrigation, rice cultivation and urbanization [14–16] whilst *An. gambiae* (*s.s.*) immatures occupy rain-dependent pools and temporary puddles [17]. Adult female of *An. gambiae* (*s.s.*), *An. coluzzii* and *An. funestus* (*s.s.*) are mostly anthropophilic and prefer resting inside human habitations, while *An. arabiensis* will feed on either humans or cattle, and rest indoors or outdoors [18].

Malaria is considered to be one of the major contributors to poverty and the estimated annual cost to economies of the African continent ranges from under 0.5 % to almost 9 % of GDP [19]. Population growth in many malaria endemic countries has led to policies advocating local food production to enable self-sufficiency in order to reduce the importation of food and to positively impact the gross national product in these countries [20–22]. The Government of Ghana recently recommended increasing local rice production through the development and/or reorganization of irrigation projects to reduce the importation of rice [23]. Though the main objective of this initiative is to cultivate and produce local rice for consumption, collateral effects are likely to be linked to the increasing resistance already detected in *An. gambiae* (*s.l.*) in these areas [24–26]. In fact, the development of irrigation schemes in sub-Saharan Africa has been blamed for the increase of malaria risk through the creation of favorable breeding sites for malaria vector mosquitoes [27–29]. Moreover, there is considerable use of herbicides, insecticides and fertilizers in irrigated rice projects; hence multiple annual rice production cycles are expected to have a dramatic impact on the insecticide resistance of malaria vectors breeding in these zones, and potentially elsewhere in the country [30, 31]. Several studies have already been conducted in Okyereko and surroundings, showing the stability of *An. coluzzii* and *An. gambiae* (*s.s.*) density throughout the whole year and its impact in terms of malaria transmission and lymphatic filariasis [32–34]. This study was carried out to investigate and to characterize the resistance mechanisms involved in the insecticide resistance of the *An. coluzzii* and *An. gambiae* (*s.s.*) from Okyereko.

## Methods

### Study site

**Fig. 1:**
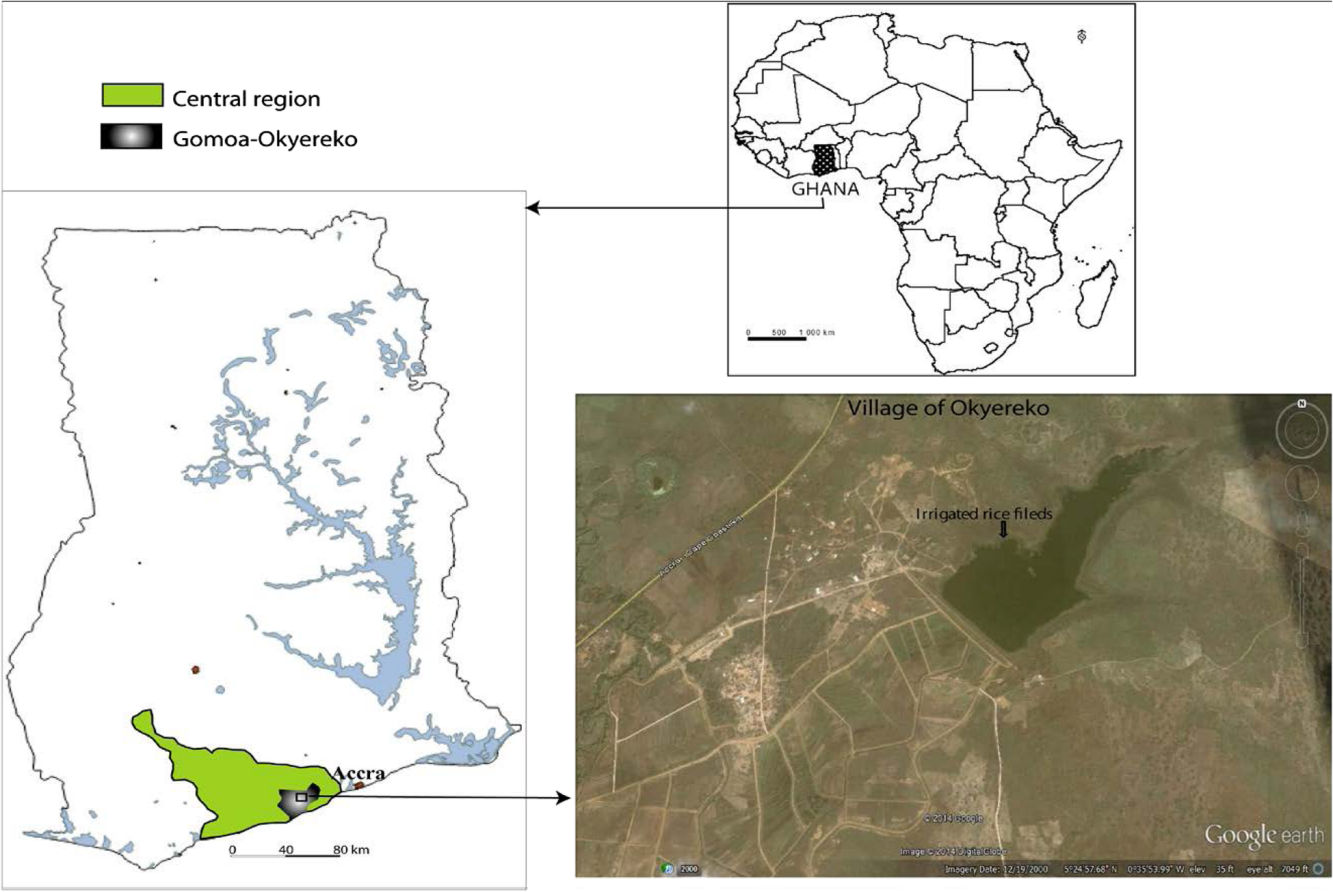
Map of the study site

Okyereko is a village located about 50 km west of Accra, the capital city of Ghana (05°24’57.68”N, 00°35’53.99”W) (Fig. 1). The climate is coastal savanna vegetation with an annual average rainfall of 750 mm. The Okyereko irrigation project consists of an earthen dam with a catchment area of about 1,685 km^2^. The reservoir is fed by the tributary of the River Ayensu. Two canals on the left and right banks of the tributary convey water to the irrigable area. Eighty-one of the 125 hectares available have been developed, including 42 ha irrigated by the project.

### Mosquito collections

Mosquito larvae were collected from water pools around the rice irrigated fields, brought to the laboratory and reared to adult stage in the insectary of Vestergaard-Noguchi Memorial Institute for Medical Research Vector Labs, Legon, in Accra. Larvae were reared in plastic trays (27 × 16 × 6.5 cm) containing 2 L of de-ionized water at a density of approximately 150/L, and fed with a mixture of finely ground tropical fish flakes. Pupae were transferred to 0.27 m^3^ screened cages and emerged adults were provided with 10 % glucose solution [35]. For consistency, all tests were conducted using the progeny of the F_1_ generation. The susceptible laboratory strain *An. gambiae* (*s.s.*) Kisumu was used as a control for all the tests that were conducted.

### WHO susceptibility tests

Mortality and knock-down resulting from tarsal contact with insecticide impregnated papers using diagnostic doses were measured using WHO susceptibility test kits [36] against *An. gambiae* (*s.l.*) females. Batches of 20–25 non-blood fed females, aged 3–5 days, were exposed to insecticide impregnated papers for 1 h for all the insecticides and 2 h specifically for fenitrothion. The numbers of mosquitoes knocked down were recorded every 10 min for 60 min for DDT and pyrethroids and after the exposure time for the other classes of insecticides. Thereafter, the mosquitoes were transferred into observation tubes, provided with pads of cotton wool soaked in 10 % glucose solution and mortality was recorded after 24 h.

Additional synergist assays were similarly performed as described above. Batches of 20–25 non blood fed females aged 3–5 days were also pre-exposed to each synergist impregnated paper for 1 h and transferred to the insecticide impregnated paper tubes for another holding period of 1 h. The number of mosquitoes knocked down and the mortality were also recorded as described above. The insecticides tested were run along with the synergist assay as positive controls. Tests with either silicone oil or olive oil impregnated papers were run in parallel and served as negative controls. The tests were performed at 25 °C ±2 °C and 70 % ± 10 % humidity.

Mosquitoes were first assayed using WHO discriminating dosages of seven insecticides belonging to four different chemical classes: deltamethrin 0.05 % and permethrin 0.75 % (pyrethroids), DDT 4 % (organochlorine), bendiocarb 0.1 % and propoxur 0.1 % (carbamates), and fenitrothion 1 % and malathion 5 % (organophosphates). Additional synergist tests were performed using Piperonyl butoxide (PBO) 5 %, Verapamil 0.1 % and S,S,S-tributylphosphorotrithioate (DEF) 0.1 %. PBO, DEF and Verapamil are known to be inhibitors of cytochrome P450s and GSTs, carboxylesterases and P-glycoprotein efflux pumps (P-gps) respectively [37–39].

Following scoring of their mortality or survival status after 24 h, all the specimens tested were preserved at −20 °C for molecular analysis. Two hundred mosquitoes from the WHO susceptibility test kept at −20 °C were randomly sampled per insecticide, sorted according to their status for DNA extraction. PCR was performed for species identification and detection of resistance mechanisms.

### Identification of sibling species in the An. gambiae complex

Genomic DNA was extracted from each mosquito using a slightly modified protocol of Collins *et al*. [40]. A single mosquito was homogenized in a 1.5 ml Eppendorf tube containing 200 μl of CTAB buffer (100 mM Tris HCL, pH 8.0, 10 mM EDTA, 1.4 M NaCl, 2 % Cetyl Trimethyl Ammonium Bromide) and incubated at 65 °C for 5 min. Chloroform (200 μl) was added and mixed by inversion of the tube. After centrifugation at 12000 rpm at room temperature for 5 min, the aqueous phase (upper layer) of the solution was pipetted into a fresh 1.5 ml tube; 200 μl of isopropyl alcohol was added, mixed by inversion and then centrifuged at 12000 rpm for 15 min. Afterwards, the supernatant was discarded and the DNA pellet formed at the bottom of tubes was washed with 70 % ethanol, dried and reconstituted in 20 μl of DNAse-free water.

Mosquito specimens were identified to the species level using the PCR method described by Scott et al. [41] and further characterized into molecular forms using the SINE PCR method of Santolamazza et al. [42]. The SINE PCR was performed using the primers; F6.1a (TCGCCTTAGACCTTGCGTTA) and R6.1b (CGCTTCAAGAATTCGAGATAC) in 25 μl PCR reaction mix containing 4 μl of 1/40th dilution of genomic DNA extracted from a single mosquito, 1 μl of 10 μM each of F6.1a and R6.1b primers, 6.5 μl of nuclease-free water and 12.5 μl of GoTaq (Promega).

### Detection of the kdr and ace-1 mutations by diagnostic PCR assays

The PCR test for the detection of *kdr* mutations was carried out as described by Martinez-Torres et al. [43, 44] and confirmed by Real Time PCR following the protocol of Bass et al. [45]. A volume of 25 μl PCR reaction consisting of 12.5 μl of GoTaq, 4.5 μl of DNase-free water, 4 μl of 1/40th dilution of DNA template and 1 μl each of 20 μM of primers AGD1 (ATAGATTCCCCGACCATG); AGD2 (AGACAAGGATGATGAACC), AGD3 (AATTTGCATTACTTACGACA) and AGD4 (CTGTAGTGATAGGAAATTTA) was prepared.

A PCR-Restriction Fragment Length Polymorphism (PCR-RFLP) diagnostic test was used to detect the presence of the *G119S* mutation in the *ace-1* gene as described by Weill et al. [46]. A mixture of 25 μl PCR reaction was prepared and this consisted of 1 μl each of 10 μM Primers EX3AGdir (GATCGTGGACACCGTGTTCG) and EX3AGrev (AGGATGGCCCGCTGGAACAG), 12.5 μl of GoTaq, 9 μl of DNase-free water and 1.5 μl of 1/40 dilution of DNA template. An enzymatic digestion step was followed after the PCR reaction. A 20 μl restriction enzyme reaction mixture was prepared, consisting of 2 μl of Enzymatic Buffer B 10X, 0.2 μl of Acetylated BSA at 10 μg/μl and 0.5 μl of 10 U/μl restriction enzyme *Alu* I (Promega), 12.3 μl of DNase-free water and 5 μl of PCR products. The mixture was incubated at 37 °C for 4 h in a thermocycler.

The resulting restriction fragments as well as all the other PCR were run on 2 % agarose gels stained with ethidium bromide and visualized under UV light.

#### Statistical analysis

The susceptibility status of the colony against each insecticide was determined following WHO criteria [47]. The population is considered resistant when mortality after 24 h is in the range of 0–90 %, suspicion of resistance is recorded mortality is between 91 and 97 %, and populations are scored as susceptible when 98–100 % of the specimens are killed.

Abbott’s formula was applied when the mortality of the controls was between 5 and 10 % [48].

The *kdr* and *ace-1* frequencies were calculated using the Hardy Weinberg formula [49] and compared with each population using the z-test for proportions with XLSTAT software.

## Results

### Resistance status

An average of 80 *An. gambiae* (*s.s.*) Kisumu strain female mosquitoes from colony were assayed for each of the seven insecticides tested and control. *Anopheles gambiae* (*s.s.*) Kisumu strain was found to be fully susceptible to all of them by yielding 100 % mortality after 24 h of observation and therefore confirmed the effectiveness of the insecticide-impregnated papers.

**Fig. 2:**
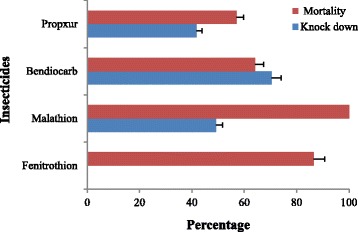
*Anopheles gambiae* (*s.l.*) female from Okyereko knock-down rate after exposure time and 24 h delayed mortality observed using organophosphate and carbamate insecticides. Error bars represent 95 % confidence interval (CI)

**Fig. 3:**
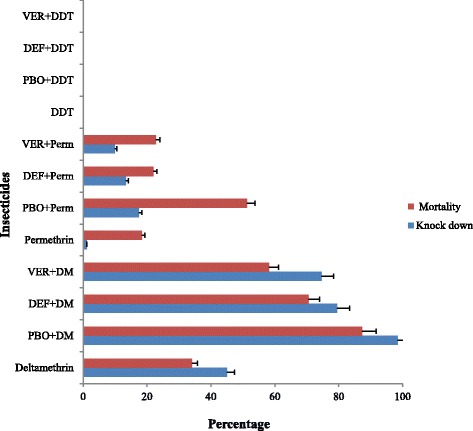
*Anopheles gambiae* (*s.l.*) female from Okyereko knock-down rate after 60 min and 24 h delayed mortality observed using DDT and Pyrethroid insecticides either alone or synergists plus insecticides. Error bars represent 95 % confidence interval (CI)

A total of 157 female *An. gambiae* (*s.l.*) from Okyereko were assayed for deltamethrin, 132 for permethrin and an average of 100 mosquitoes for all other insecticides. Mortality observed with DDT and pyrethroid insecticides was very low, ranging from 0 % with DDT to 18.4 and 34.1 % with permethrin and deltamethrin respectively. Higher mortalities were observed with the organophosphates with 86 % mortality for fenitrothion and 100 % with malathion. The observed mortalities using carbamates were 64 % for bendiocarb and 57 % for propoxur (Figs. 2 and 3).

Mortality with pyrethroids was slightly improved with pre exposure to synergists. PBO showed the highest improvement with mortality increasing by more than 2.5 fold for deltamethrin and permethrin. DEF + permethrin and Verapamil + permethrin did not show any significant mortality increment compared to permethrin alone (*P* > 0.05). A significantly higher increase in mortality was observed with PBO + deltamethrin than with PBO + permethrin, as well with DEF and Verapamil to synergize both pyrethroid insecticides. DEF and Verapamil yielded 2 and 1.7 fold increases in mortality rates with deltamethrin, respectively. No increase in mortality was observed with pre exposure to synergists and DDT (Fig. 3).

### Identification of An. gambiae complex species and characterization of the resistance mechanisms

Two hundred mosquitoes were identified as *An. gambiae* (*s.l.*) of which 193 were *An. coluzzii*, representing 96.5 % and the remaining seven were identified as *An. gambiae* (*s.s.*) Allele and genotype frequency results at the *kdr* and *ace-1* loci of *An. gambiae* (*s.l.*) (Table 1), showed a high prevalence of the *kdr L1014F* mutation in the *An. gambiae* populations at Okyereko at a frequency of almost 90 %. Most of the mosquitoes tested were either homozygotes (RR) or heterozygotes (RS). Only four homozygote susceptible (SS) mosquitoes were found among the analyzed samples. Secondly, both forms showed similar frequency of 0.9, though the number of *An. coluzzii* was far larger than the number of *An. gambiae* (*s.s.*).

**Table 1:** Frequency of species, genotype of *kdr L1014F* and *ace-1 G119S* of *An. coluzzii* and *An. gambiae* (*s.s.*) from Okyereko

Total analyzed	Species		*Kdr* mutation		Freq/species	Ace-1 mutation		Freq/species
200	*An. coluzzii*	193 (96.5 %)	RR	143	0.9	RR	0	0.2
			RS	46		RS	70	
			SS	4		SS	123	
	*An. gambiae* (*s.s.*)	7 (3.5 %)	RR	5	0.9	RR	0	0.3
			RS	2		RS	4	
			SS	0		SS	3	

The *ace-1* detected in both *An. coluzzii* and *An. gambiae* (*s.s.*) were at a very low frequency. Only few heterozygote RS genotypes were observed giving a frequency of 2 % *ace-1* mutation. Moreover, the frequency of *ace-1* observed among both species was not significantly different (*p* = 0.433) with 0.2 and 0.3 % in *An. coluzzii* and *An. gambiae* (*s.s.*), respectively.

The presence of the *kdr* and *ace-1* allele was also compared between the dead and the surviving mosquitoes after the WHO susceptibility testing. No significant difference in the *kdr* distribution between the two groups was observed (*P* = 0.699). In contrast, there was a significantly higher rate of RS genotypes among the mosquitoes that survived than among the dead specimens (*P* < 0.0001) for the *ace-1* mutation (Table 2). The frequency of the *ace-1* mutation observed was 0.25 for the surviving mosquitoes and significantly higher than that recorded in the dead mosquitoes (0.11) (*P* < 0.009).

**Table 2:** Comparison of the frequency of the *kdr* and *ace-1* mutations following the phenotypic status (dead/alive) of *An. gambiae* s.l. from Okyereko after WHO susceptibility testing

Insecticides	Mosquito status	Number tested	*kdr* genotype			*kdr* freq/status	*P-value* dead/alive *kdr* freq	Number tested	*ace-1* genotype			*ace-1* freq/status	*p value* dead/alive *ace-1* freq
			RR	RS	SS				RR	RS	SS		
Deltamethrin	Alive	25	17	8	0	-	***0.699***	25	0	7	18	-	***0.009***
Permethrin		25	17	7	1	-		25	0	7	18	-	
DDT		25	19	5	1	-		25	0	7	18	-	
Bendiocarb		14	11	3	0	-		14	0	14	0	-	
Propoxur		16	14	2	0	-		16	0	15	1	-	
Fenitrothion		6	6	0	0	-		6	0	6	0	-	
**Total tested**		**111**	**84**	**25**	**2**	**0.87**		**111**	**0**	**56**	**55**	**0.25**	
Deltamethrin	Dead	3	3	0	0	-		3	0	1	2	-	
Permethrin		2	2	0	0	-		2	0	0	2	-	
Bendiocarb		17	13	4	0	-		17	0	1	16	-	
Propoxur		18	14	4	0	-		18	0	1	17		
Fenitrothion		18	12	6	0	-		18	0	4	14	-	
Malathion		26	15	9	2	-		26	0	12	14		
**Total tested**		**84**	**59**	**23**	**2**	**0.84**		**84**	**0**	**19**	**65**	**0.11**	
Control		5	5	0	0	1		5	0	2	3	0.2	

The bold texts represent the specifications of the total number of specimen tested per mosquito status, either dead or alive. The numbers in bold represent the total numbers and genotypes of the total number of the mosquitoes tested per status

## Discussion

More than 96 % of the mosquitoes collected from Okyereko rice field area were identified as *An. coluzzii,* similar to species ratios reported in previous studies [32, 34]. This agrees with previous observations suggesting that *An. coluzzii* is more adapted to breed in irrigated fields, in contrast to *An. gambiae* (*s.s.*), which is described as a species of humid forested areas and temporary pools [50, 51].

The long-term use of insecticides in agriculture and households has been implicated in the increasing insecticide resistance of insect vectors and particularly the malaria vector *An. gambiae* (*s.l.*) [1, 5, 9, 52–55]*.* This study demonstrated that mosquito populations from Okyereko are resistant to pyrethroid, organochlorine and carbamate classes of insecticides, as well as an organophosphate commonly used in vector control programmes. Insecticide resistance mechanisms of *An. coluzzii* and *An. gambiae* (*s.s.*) from Okyereko involved high levels of *kdr L1014F* mutation, mainly expressed in resistance to DDT and pyrethroids, with 0 % mortality against DDT and less than 35 % for permethrin and deltamethrin. The use of PBO, in addition to either permethrin or deltamethrin increased the mortality rates by 2× and 3× respectively, indicating the likely involvement of oxidases and potentially esterase-based metabolic resistance mechanisms. Although oxidases may be involved in the detoxification of almost all insecticides, they are known to be mostly associated with pyrethroid resistance [38]. Furthermore, there are increasing reports of oxidases being involved in the metabolism of carbamates [56], which likely contributed to the higher carbamate resistance level detected than organophosphate. No effects of synergists were found with DDT, which is likely affected by the *kdr* mutation and possibly Glutathione-S-Transferase (GST) mechanisms, although increased GST activity was not verified in this study [57, 58]. The DDT mortality observed were similar to the extremely high resistance reported by Chouaibou *et al.* [59], in *An. gambiae* (*s.l.*) collected from rice irrigation fields of Tiassalé in Côte d’Ivoire.

These results also show a significant increase in mortality to deltamethrin after a pre-exposure to verapamil, suggesting a potential role of P-gps in the resistance mechanisms to this pyrethroid [37]. Several studies have demonstrated that pesticides in mammalian cells were partially regulated through the action of P-gps [60, 61]. These proteins belong to the superfamily of ATP-Binding Cassette (ABC transporters), which pump molecules out of cells by an ATP-dependent mechanism [62] and have been associated with resistance to a number of drugs [63]. The role of P-gps in defense against temephos and diflubenzuron insecticides in *Aedes caspius* (Pallas) has been demonstrated using Verapamil [64], so similar mechanisms in other species of mosquitoes such as *An. gambiae* (*s.l.*) are not unlikely.

The use of DEF, inhibitor of carboxylesterase activity yielded similar results as Verapamil for all the insecticides that were synergized. Significant improvement was noted with deltamethrin and permethrin. Carboxylesterase is involved in the detoxification of various chemicals, including pesticides. Inhibition of carboxylesterase by DEF has resulted in the potentiation of organophosphate pesticides such as Malathion that contain a carboxylic ester group [39]. Nevertheless, this trend wasn’t tested in this study due to the susceptibility level already expressed by the population, showing 100 and 86 % mortality against malathion and fenitrothion respectively.

Both *An. coluzzii* and *An. gambiae* (*s.s.*) expressed high pyrethroid-resistance with the involvement of the *kdr L1014F* mutation at almost the same frequency. No correlation was observed between the phenotypic status of the mosquitoes after the susceptibility test and the genotype of the specimens [65]. In contrast, the *ace-1 G119S* mutation was observed in a very low proportion among the dead mosquitoes. Only a few heterozygous mosquitoes were found after analysis (Freq_*ace-1*_ = 0.1). A correlation between the life status of the mosquitoes after WHO tube testing and the *ace-1* mutation genotype was noted, with most of the dead mosquitoes found to be susceptible, and the majority of heterozygous RS found among those surviving. The correlation of the life status of mosquitoes has been well described with the carbamate insecticides where 75 % of heterozygous resistant (RS) were alive and 77 % of the dead mosquitoes were homozygous susceptible (SS). A similar trend was also observed using *An. gambiae* (*s.l.*) mosquitoes from Tiassalé in Côte d’Ivoire by Edi *et al*. [66], where the *ace-1* mutation was mostly expressed among the mosquitoes surviving from the WHO susceptibility test. Overall, the insecticide resistance level of the mosquitoes from Okyereko could lead to a failure of some vector control measures in the area. It is well demonstrated that the ineffectiveness of LLINs impregnated with pyrethroid insecticides was mostly linked to the high *kdr* allele frequency of *An. gambiae* (*s.l.*) [9, 67].

## Conclusion

This study brings additional information on the resistant status of the *An. coluzzii* and *An. gambiae * (*s.s.*) from Okyereko. The implications of this resistance profile for malaria control are considerable and should be taken into account when considering insecticide choices in the vector control programmes in the region. A policy for controlling insecticide use in irrigated fields would likely help slowdown the development of insecticide resistance in these areas.
